# Top Concerns of Tweeters During the COVID-19 Pandemic: Infoveillance Study

**DOI:** 10.2196/19016

**Published:** 2020-04-21

**Authors:** Alaa Abd-Alrazaq, Dari Alhuwail, Mowafa Househ, Mounir Hamdi, Zubair Shah

**Affiliations:** 1 College of Science and Engineering Hamad Bin Khalifa University Doha Qatar; 2 College of Life Sciences Kuwait University Kuwait Kuwait; 3 Health Informatics Unit Dasman Diabetes Institute Kuwait Kuwait

**Keywords:** coronavirus, COVID-19, SARS-CoV-2, 2019-nCov, social media, public health, Twitter, infoveillance, infodemiology, health informatics, disease surveillance

## Abstract

**Background:**

The recent coronavirus disease (COVID-19) pandemic is taking a toll on the world’s health care infrastructure as well as the social, economic, and psychological well-being of humanity. Individuals, organizations, and governments are using social media to communicate with each other on a number of issues relating to the COVID-19 pandemic. Not much is known about the topics being shared on social media platforms relating to COVID-19. Analyzing such information can help policy makers and health care organizations assess the needs of their stakeholders and address them appropriately.

**Objective:**

This study aims to identify the main topics posted by Twitter users related to the COVID-19 pandemic.

**Methods:**

Leveraging a set of tools (Twitter’s search application programming interface (API), Tweepy Python library, and PostgreSQL database) and using a set of predefined search terms (“corona,” “2019-nCov,” and “COVID-19”), we extracted the text and metadata (number of likes and retweets, and user profile information including the number of followers) of public English language tweets from February 2, 2020, to March 15, 2020. We analyzed the collected tweets using word frequencies of single (unigrams) and double words (bigrams). We leveraged latent Dirichlet allocation for topic modeling to identify topics discussed in the tweets. We also performed sentiment analysis and extracted the mean number of retweets, likes, and followers for each topic and calculated the interaction rate per topic.

**Results:**

Out of approximately 2.8 million tweets included, 167,073 unique tweets from 160,829 unique users met the inclusion criteria. Our analysis identified 12 topics, which were grouped into four main themes: origin of the virus; its sources; its impact on people, countries, and the economy; and ways of mitigating the risk of infection. The mean sentiment was positive for 10 topics and negative for 2 topics (deaths caused by COVID-19 and increased racism). The mean for tweet topics of account followers ranged from 2722 (increased racism) to 13,413 (economic losses). The highest mean of likes for the tweets was 15.4 (economic loss), while the lowest was 3.94 (travel bans and warnings).

**Conclusions:**

Public health crisis response activities on the ground and online are becoming increasingly simultaneous and intertwined. Social media provides an opportunity to directly communicate health information to the public. Health systems should work on building national and international disease detection and surveillance systems through monitoring social media. There is also a need for a more proactive and agile public health presence on social media to combat the spread of fake news.

## Introduction

Since the 1980s, human disease outbreaks have become increasingly frequent and diverse due to a plethora of ecological, environmental, and socioeconomic factors [1]. The family of coronaviruses was not considered to be highly pathogenic until 2003 and 2012 with the appearance of the severe acute respiratory syndrome in China followed by the Middle East respiratory syndrome in Saudi Arabia [2,3]. In December 2019, a series of patients with pneumonia of an unknown cause emerged in Wuhan, China [4]. Through contact tracing, these patients were linked back to a seafood and wet animal wholesale market in Wuhan [4]. To further investigate the symptoms, Chinese authorities conducted deep sequence analysis that provided ample evidence that the novel coronavirus was the causative agent of the disease [4], which is now known as the coronavirus disease (COVID-19). Since then, COVID-19 has quickly spread in China and other countries around the world. The disease is highly infectious, and, on average, each patient can spread the infection from 2 to 4 other individuals [5]. Worldwide, a total of 1,279,722 cases of COVID-19 and 72,614 deaths were confirmed in 212 countries by April 7, 2020 [6].

With the worldwide spread of the COVID-19 infection, individual activity on social media platforms such as Facebook, Twitter, and YouTube began to increase. A number of studies have shown that social media can play an important role as a source of data for detecting outbreaks but also in understanding public attitudes and behaviors during a crisis as a way to support crisis communication and health promotion messaging [7-11]. To assist public health professionals to make better decisions and aide their public health monitoring, advanced surveillance systems are developed to sort through large amounts of real time data from social media concerning public health information on a global scale [7]. Publicly accessible data posted on social media platforms by users around the world can be used to quickly identify the main thoughts, attitudes, feelings, and topics that are occupying the minds of individuals in relation to the COVID-19 pandemic. Such data can help policymakers, health care professionals, and the public identify primary issues that of concern and address them in a more appropriate manner.

A growing body of literature has been centered on examining the use of Twitter for public health research. A systematic review paper identified six main uses of Twitter for public health: analysis of shared content, surveillance of public health topics or diseases, public engagement, recruitment of research participants, Twitter-based public health interventions, and network analysis of Twitter users [9]. Other studies analyzed twitter data for sentiment analysis [12] and the use of Twitter to propagate credible vaccine-related web pages [8]. Building on previous work, this study aims to identify the main topics posted by Twitter users related to the COVID-19 pandemic. Analyzing such information can help policy makers and health care organizations assess the needs of their stakeholders and address them in an appropriate and relevant manner.

## Methods

### Data Collection

We collected coronavirus-related tweets between February 2, 2020, and March 15, 2020, using the Twitter standard search application programming interface (API) consisting of a set of predefined search terms (“corona,” “2019-nCov,” and “COVID-19”), which are the most widely used scientific and news media terms relating to the novel coronavirus. We extracted and stored the text and metadata of the tweets using the time stamp, number of likes and retweets, and user profile information including the number of followers. We stored the tweets in a database table, where the primary key of the table was tweet ID. As a result, the duplicates were not stored in our database. Only English language tweets were collected in the study. Since the metadata of tweets such as the number of likes and retweets might change over time, we recollected the updated metadata of the tweets at the end of the study period using the tweet IDs of the already collected tweets. Twitter standard search API allows the access of old tweets using tweet IDs. We used the Tweepy Python (Python Software Foundation) library for accessing the Twitter API and PostgreSQL (PostgreSQL Global Development Group) database for storing the collected tweets.

### Data Preprocessing

**Figure 1:**
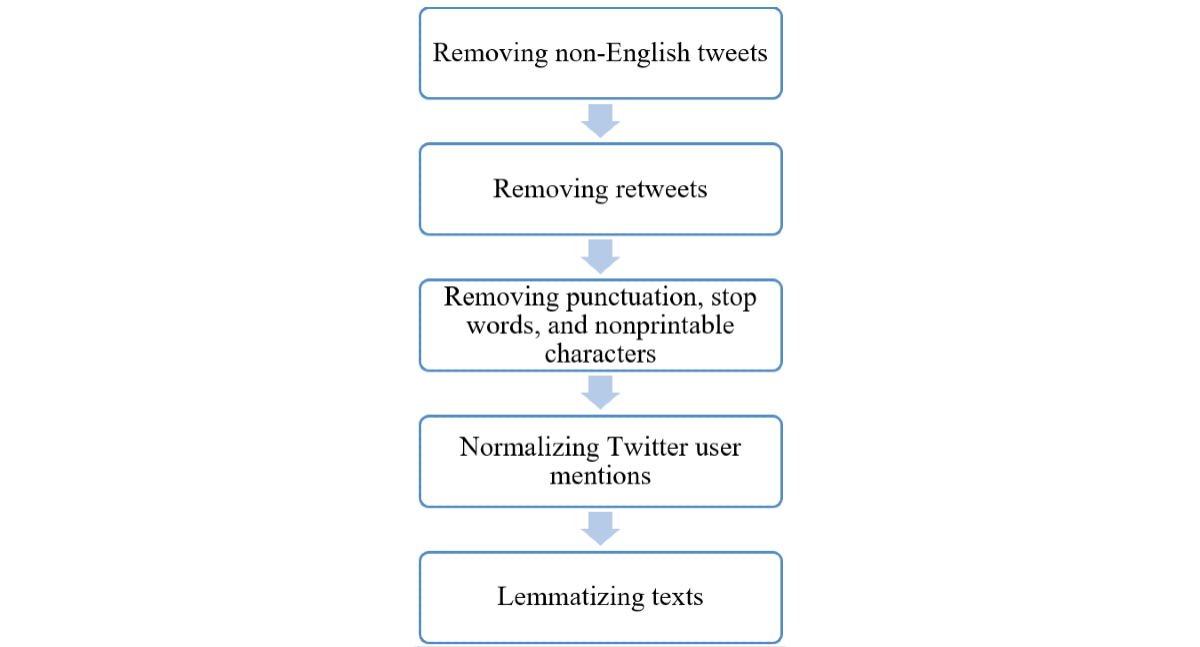
Data preprocessing workflow.

We identified non-English tweets using the language field in the tweets metadata and removed them from the analysis. We identified and removed retweets from the analysis. We also removed punctuation, stop words such as *an* and *the*, and nonprintable characters such as emojis from the tweets. We normalized Twitter user mentions by converting, for example, “@Alaa” to “@username.” Furthermore, various forms of the same word (eg, travels, traveling, and travel’s) were lemmatized by converting them to the main word (eg, travel) using the WordNetLemmatizer module of the Natural Language Toolkit Python library. The data preprocessing is depicted in Figure 1. Following the terms and conditions, terms of use, and privacy policies of Twitter, all data were anonymized and were not reported verbatim to any third party.

### Data Analysis

The processed tweets were analyzed using word frequencies of single words (unigram) and double-word (bigrams) combinations, and they were visualized through word clouds to identify the most common topics. In addition, we used the topic modeling technique [13] to identify the most common topics in the tweets. Topic modeling is an unsupervised machine learning technique that can find clusters in a collection of documents (tweets in this case). We used the latent Dirichlet allocation (LDA) algorithm from the Python sklearn package. LDA requires a fixed set of topics, where each topic is represented by a set of words. The objective of LDA is to map the given documents to the set of topics so that the words in each document are mostly captured by those topics. LDA is a widely used topic modeling algorithm. We used it to find natural clusters in the language of tweets. We applied topic modeling by specifying the number of topics required by the LDA to separate the set of tweets into various clusters. Based on our previous work, we selected 30 to be the number of topics for running the LDA [14].

We took the top representative words of each of the 30 topics produced by the LDA topic modelling algorithm (see LDA output in Multimedia Appendix 1) and the common words from the word cloud (see word cloud in Multimedia Appendix 2) and manually analyzed both sets of words. From this manual analysis, the authors reached a consensus on 12 topics and associated terms, unigram and bigram, for each topic (see associated terms for each topic in Multimedia Appendix 3). These terms were used to classify tweets, using a *rule-based classification script*, into different topics and compute the prevalence of each topic.

Next, we developed a rule-based classification script written in Python to check for the presence of any of the preidentified unigrams and bigrams in each tweet. The classification script used a simple string-matching technique to see if a given tweet contains the selected keywords of the topics. A tweet that contained a selected keyword related to a certain topic was classified as belonging to that topic.

We also performed other analyses such as sentiment analysis, which extracts the mean number of retweets, likes, and followers for each topic and then calculates the interaction rate for each topic. The sentiment analysis was performed on the tweet text using the Python textblob library. The sentiment score varied between –1.0 to 1.0, with –1.0 as the most negative text and 1.0 as the most positive text. We calculated the mean sentiment and the mean number of likes, retweets, and followers for each topic. We also calculated the interaction rate for each topic by summing the total number of retweets and likes per topic divided by the sum of the total number of followers per topic. These measures provided additional insight into the topics and users who posted in these topics.

## Results

### Search Results

**Figure 2:**
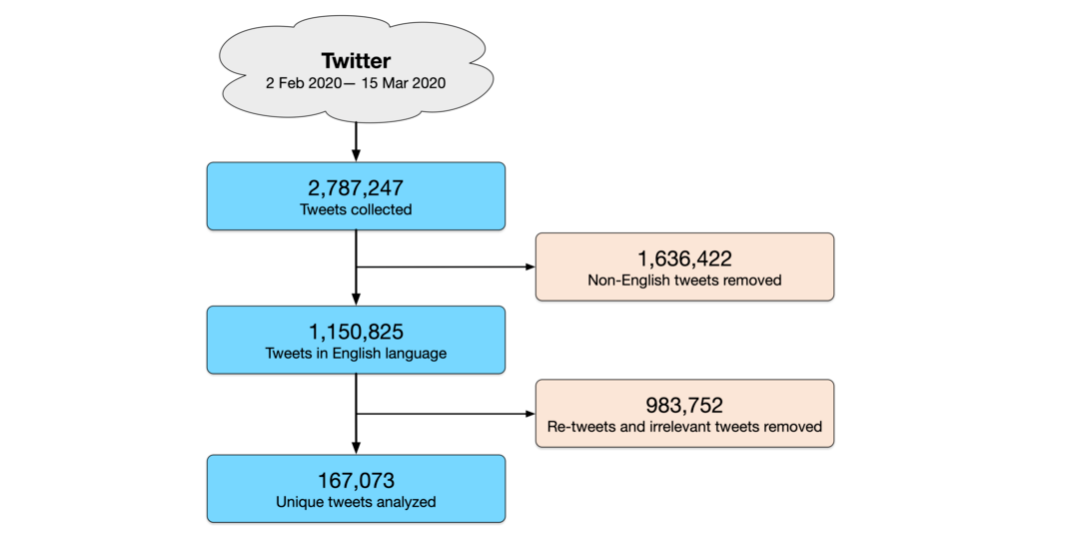
Flowchart of selection of tweets.

As shown in Figure 2, a total of 2,787,247 tweets were obtained between February 2, 2020, and March 15, 2020. Of these tweets, 1,636,422 (58.71%) non-English tweets were removed. Of the 1,150,825 remaining English tweets, 735,182 ‬(63.88%) retweets were excluded. A further 248,570 (21.60%) tweets with no coronavirus-related terms in the text were also removed. These tweets were captured by Twitter API either because the name or the profile description of users matched the search terms. Accordingly, the study analyzed 167,073 unique tweets from 160,829 unique users.

### Results of Tweet Analysis

#### Topics Emerged From Tweets

We identified 12 topics from the analyzed tweets. The 12 topics were grouped into four themes: the origin of COVID-19, the source of a novel coronavirus, the impact of COVID-19 on people and countries, and the methods for decreasing the spread of COVID-19. Table 1 summarizes the prevalence of the identified topics. Values on the diagonal of the table refer to numbers and percentages of tweets in a topic, and values in the off-diagonal of the table indicate numbers and percentages of tweets in the intersection of the two topics. For instance, a hypothetical tweet such as “while the death toll due to COVID-19 continues to rise, the travel ban imposed by countries to limit the spread of coronavirus infection started to affect the daily life of many people” could be classified under travel and death. The value at the intersection for these 2 topics in the table represents the number and percentage of tweets containing keywords related to both topics. More details about themes in these topics are elaborated in the following subsections.

##### Theme 1: Origin of COVID-19

This theme contains two topics that discuss the origin of COVID-19. The first topic was China, which was the most common topic of all identified topics. Tweeters talked about China as it was the country where the novel coronavirus originated from. The second topic was the outbreak. The tweets in this topic talked about the details of the outbreak, such as how, when, and where the outbreak emerged.

##### Theme 2: Source of the Novel Coronavirus

This theme included tweets about the causes leading to the transfer of COVID-19 to humans. Tweeters identified two sources of a novel coronavirus, which formed two topics in this study: eating meat and developing bioweapons. The former topic (eating meat) was identified in tweets mentioning the role of meat in the spread of COVID-19. Most of these tweets blamed nonvegetarians for the outbreak of COVID-19 and asked them to stop eating meat to stop the coronavirus spread. The latter topic (bioweapon) was formed by the tweets of individuals debating whether or not the COVID-19 virus originated from a Chinese biological military laboratory.

##### Theme 3: Impact of COVID-19 on People and Countries

The third theme was generated from tweets about the influence of COVID-19 on people, companies, and countries. The tweets in this theme identified six effects of COVID-19, which also formed six topics. The first topic related to the number of deaths caused by COVID-19. The tweets that belonged to this topic mainly showed statistics and numbers of deaths caused by a coronavirus in different cities and countries.

The second topic was the fear and stress caused by COVID-19. Twitter users in these tweets expressed their fear and stress about the coronavirus due to its quick spread and the lack of treatments or vaccines for the disease caused by the coronavirus.

The third topic was related to the effects of COVID-19 on travel from and to China and other countries. These tweets mostly discussed flight cancellations, postponements, travel bans, and restrictions as well as travel warnings imposed by many countries due to the coronavirus pandemic.

The impact of COVID-19 on the economy was the fourth topic. These tweets mostly showed actual or expected losses in the economy of many companies and countries due to, for example, closure of markets, a decrease of oil demands, delays in production, and canceling of important events, which came as a result of the COVID-19 outbreak.

Panic buying was the fifth topic identified. These tweets talked about how individuals in many countries became panic buyers in preparation for curfews, lockdowns, and stay-at-home orders due to the COVID-19 pandemic, and how supermarkets and shops controlled and prevented panic buying.

The last topic identified in this theme related to racism. Specifically, users in most of the tweets reported the spreading of racist, prejudiced, and xenophobic attacks (eg, rude comments or dirty looks) against East Asians given that COVID-19 originated from their countries.

##### Theme 4: Methods for Decreasing the Spread of COVID-19

The last theme brought together tweets that discussed methods for decreasing the spread of COVID-19. Two methods were identified from these tweets and formed the following two topics: wearing masks and the quarantine of people. Most of the tweets from the former topic talked about either the importance of face masks in decreasing the outbreak of the coronavirus or their shortage in several countries. Most of the tweets from the latter topic were about quarantining individuals who were infected with or suspected to have the coronavirus to reduce or prevent the spread of the disease.

As shown in the off-diagonal values in Table 1, the most common topic overlap was between China and deaths caused by COVID-19, followed by China and eating meat, China and the outbreak of COVID-19, deaths caused by COVID-19 and eating meat, and China and fear and stress about COVID-19.

**Table 1:** Numbers and percentages of tweets (N=167,073) related to each topic (diagonal values) and at the intersection of two topics (off-diagonal values).

Themes and subtopics		China, n (%)		Outbreak of COVID-19^a^, n (%)		Eating meat, n (%)		Developing bioweapon, n (%)		Deaths caused by COVID-19, n (%)		Fear and stress about COVID-19, n (%)		Travel bans and warnings, n (%)		Economic losses, n (%)		Panic buying, n (%)		Increased racism, n (%)		Wearing masks, n (%)		Quarantining subjects, n (%)	
**Origin of COVID-19**																									
	China		27,128 (16.24)		—^b^		—		—		—		—		—		—		—		—		—		—
	Outbreak of COVID-19		2776 (1.66)		7468 (4.47)		—		—		—		—		—		—		—		—		—		—
**Source of novel coronavirus**																									
	Eating meat		4200 (2.51)		560 (0.34)		12,772 (7.65)		—		—		—		—		—		—		—		—		—
	Developing bioweapon		808 (0.48)		151 (0.09)		220 (0.13)		2021 (1.21)		—		—		—		—		—		—		—		—
**Impact of COVID-19 on people and countries**																									
	Deaths caused by COVID-19		4332 (2.59)		905 (0.54)		2621 (1.57)		219 (0.13)		17,606 (10.54)		—		—		—		—		—		—		—
	Fear and stress about COVID-19		1820 (1.09)		484 (0.29)		841 (0.50)		137 (0.08)		1421 (0.85)		8785 (5.26)		—		—		—		—		—		—
	Travel bans and warnings		912 (0.55)		424 (0.25)		175 (0.10)		25 (0.01)		313 (0.19)		339 (0.20)		4358 (2.61)		—		—		—		—		—
	Economic losses		1019 (0.61)		273 (0.16)		208 (0.12)		65 (0.04)		192 (0.11)		198 (0.12)		67 (0.04)		2565 (1.54)		—		—		—		—
	Panic buying		598 (0.36)		175 (0.10)		115 (0.07)		39 (0.02)		183 (0.11)		161 (0.10)		83 (0.05)		826 (0.49)		2161 (1.29)		—		—		—
	Increased racism		614 (0.37)		98 (0.06)		134 (0.08)		7 (0.01)		191 (0.11)		192 (0.11)		32 (0.02)		9 (0.01)		22 (0.01)		2136 (1.28)		—		—
**Methods for decreasing COVID-19 spread**																									
	Wearing masks		560 (0.34)		221 (0.13)		166 (0.10)		16 (0.01)		293 (0.18)		218 (0.13)		113 (0.07)		50 (0.03)		178 (0.10)		51 (0.03)		3397 (2.03)		—
	Quarantining subjects		524 (0.31)		148 (0.09)		90 (0.05)		15 (0.01)		251 (0.15)		134 (0.08)		322 (0.19)		32 (0.02)		20 (0.01)		12 (0.01)		39 (0.02)		2014 (1.21)

^a^COVID-19: coronavirus disease.

^b^—: not available.

### Results of Sentiment and Interaction Rate Analysis

As shown in Table 2, the mean of sentiment was positive in all topics except two: deaths caused by COVID-19 and increased racism. The highest mean of positive sentiments was for the eating meat topic, followed by the wearing masks topic. The highest mean of negative sentiments was for “deaths caused by COVID-19” topic.

**Table 2:** Results of sentiment and interaction analysis for tweets (N=167,073).

Topics	Sentiment, mean (SD)	Followers, mean (SD)	Likes, mean (SD)	Retweets, mean (SD)	Interaction rates	User mentions, n (%)	Link sharing, n (%)
China	0.028 (0.254)	5971.83 (182,938.26)	5.48 (128.42)	1.65 (51.08)	0.00120	10,323 (6.18)	11,041 (6.61)
Outbreak	0.037 (0.229)	20,498.22 (272,064.16)	6.48 (88.02)	2.69 (50.75)	0.00045	2038 (1.23)	3090 (1.85)
Eating meat	0.082 (0.282)	7177.12 (176,101.49)	12.34 (295.47)	7.09 (136.75)	0.00271	3815 (2.28)	7140 (4.27)
Developing bioweapon	0.016 (0.241)	3071.80 (22,697.08)	6.66 (114.81)	2.24 (37.53)	0.00290	1036 (0.62)	706 (0.42)
Deaths caused by COVID-19^a^	–0.057 (0.287)	9020.53 (204,289.34)	6.00 (86.42)	2.44 (39.75)	0.00094	6847 (4.10)	5924 (3.55)
Fear and stress about COVID-19	0.015 (0.247)	11,755.66 (310,842.61)	7.11 (129.05)	2.42 (48.22)	0.00081	3851 (2.30)	2693 (1.61)
Travel bans and warnings	0.032 (0.248)	9003.54 (154,933.20)	3.93 (33.27)	0.92 (8.07)	0.00054	2122 (1.27)	1210 (0.72)
Economic losses	0.035 (0.247)	13,361.82 (287,310.56)	15.33 (517.00)	3.58 (109.51)	0.00141	1225 (0.73)	846 (0.51)
Panic buying	0.031 (0.248)	12,121.17 (456,517.30)	4.07 (38.95)	0.89 (8.51)	0.00041	944 (0.56)	609 (0.36)
Increased racism	–0.033 (0.264)	2878.38 (64,604.27)	9.87 (80.57)	1.66 (14.89)	0.00400	685 (0.41)	427 (0.26)
Wearing masks	0.035 (0.262)	7557.34 (147,010.30)	8.08 (105.39)	1.88 (28.68)	0.00132	1200 (0.72)	1062 (0.64)
Quarantining subjects	0.012 (0.263)	6800.47 (87835.42)	5.64 (39.10)	1.90 (17.12)	0.00111	896 (0.54)	630 (0.38)

^a^COVID-19: coronavirus disease.

The mean of followers for tweeters who posted the collected tweets ranged from 2878 (in increased racism) to 13,361 followers (in economic losses). The economic loss topic had the highest mean of likes. On the other hand, travel ban and warning-related topics had the lowest mean of likes. The mean of retweets for the collected tweets varied between 0.89 (for panic buying) and 7.11 (for eating meat). The lowest interaction rate was for panic buying–related tweets, and the highest interaction rate was for racism-related tweets followed by bioweapon-related tweets and eating meat–related tweets (Table 2).

User mentions were the most common in China-related tweets, but they were the least common in racism-related tweets (Table 2). Similarly, link sharing was the most common in China-related tweets, whereas they were the least common in racism-related tweets (Table 2). Multimedia Appendix 4 shows more descriptive statistics (ie, medians, variances, standard deviations, maximums, and minimums) for all previously mentioned measures.

## Discussion

### Principal Findings

Users on Twitter discussed 12 main topics across four main themes related to COVID-19 between February 2, 2020, and March 15, 2020. User mentions and link sharing were the most common in the analyzed tweets. These findings might demonstrate that users on Twitter are interested in notifying or warning their friends and followers about COVID-19. These interpersonal communications indicate that people bond around the topic of COVID-19 on Twitter.

Users on Twitter also focused on the impact of coronavirus on people and countries. Specifically, numerous tweets were posted on the number of deaths linked to the coronavirus. Furthermore, the emotional and psychological impact of the coronavirus was mentioned in many tweets. Users on Twitter may show their fear and stress about COVID-19 and the lack of vaccine treatment options to prevent it or specific antiviral treatments [15]. However, the sensationalistic use of Twitter can be a great challenge for public health and outbreak response efforts because of the wild spread of misinformation and conspiracy theories [16]. The infectious outbreak of “fake news” and “distorted evidence” in the digital world can create mass panic and cause damaging and devastating consequences in the real world, distorting evidence and impeding the response efforts and activities of health care workers and public health systems [17].

Additionally, the economic impact of COVID-19 on companies and countries were discussed in several tweets. Tweeters might talk about the economic impact of COVID-19 due to, for example, temporary closures of major fast-food chains and retailers (eg, McDonald’s, KFC, Apple, and Adidas) [18], decreases in auto sales, drops in oil demand, production delays such as with the iPhone, the canceling or postponing of sporting events such as the Formula One World Championship, or decreases in airline revenues due to flight cancellations [18,19]. It has been estimated that the spread of COVID-19 could cost the worldwide economy a total of US $2.7 trillion [20]. The last impact of COVID-19 discussed by Twitter users was travel. This topic might have been common because most countries have banned travel from and to countries that confirmed the presence of COVID-19 inside their borders.

Tweets also focused on two possible sources of the coronavirus: the eating of meat and a Chinese biological military laboratory. Tweeters mentioned two main methods used to decrease the spread of COVID-19: masks and quarantine. The first method (masks) was discussed frequently on Twitter mainly due to the face mask shortage reported in several countries (eg, China, the United Kingdom, and the United States). The quarantine was a common topic in tweets because it was the first step that countries applied to control the outbreak of COVID-19.

### Practical and Research Implications

#### Practical Implications

Research shows that crisis response activities in reality and online are becoming increasingly “simultaneous and intertwined” [21]. Social media provides a lucrative opportunity to spread and disseminate public health knowledge and information directly to the public [22]. However, social media can also be a powerful weapon and, if not used appropriately, can be destructive to public health efforts, especially during a public health crisis.

Therefore, more efforts are needed to build national and international detection and surveillance systems of diseases by examining online content published through the World Wide Web, including social media. There is a need for stronger and more proactive public health presence on social media. Governments and health systems should also “listen” or monitor the tweets from the public that relate to health, especially in a time of crisis, to help inform policies related to public health (eg, social distancing and quarantine) and supply chains among many others.

#### Research Implications

The global COVID-19 outbreak and its wild spread across countries demonstrates the need for more vigilant and timely responses aided by the research community. This was not the focus of this study, but future studies should investigate the spread of “fake news” in combination with infectious disease outbreaks [23]. Moreover, there is a need for providing access to a core corpus of social media posts available to the scientific and public health community while maintaining privacy. Additional work is necessary for multilingual sentiment analysis on social media platforms, as most research efforts have been devoted to English-language data [24], including this study. It could also be useful for future studies to consider longitudinal, multilingual sentiment analysis in addition to concurrent analysis of infectious disease outbreaks on different social media platforms, if feasible.

### Strengths and Limitations

Several strengths and limitations can be attributed to this study analyzing tweets related to the recent COVID-19 outbreak. In this study, no geographical restrictions were applied on the tweets analyzed considering the worldwide spread of the disease. However, the study only analyzed tweets in the English language, which may limit the generalizability of the findings about this worldwide outbreak. In addition, given that the Twitter standard search API does not allow researchers to obtain tweets posted more than 1 week ago [25], we could not get COVID-19-related tweets posted before February 2, 2020. Thus, the findings may not be generalizable to that period. Moreover, this study could not collect tweets from accounts marked as private. Therefore, findings may not represent all the topics discussed by users on Twitter related to COVID-19. Only posts on Twitter were analyzed in this study, thereby, our findings may not be generalizable to other social media platforms. Furthermore, the findings reported in this study are limited to only those that have access to and use Twitter. Therefore, caution is advised before assuming the generalizability of the results, as Twitter is not used by everyone in the population.

### Conclusion

The COVID-19 pandemic has been affecting many health care systems and nations, claiming the lives of many people. As a vibrant social media platform, Twitter projected this heavy toll through the interactions and posts people made related to COVID-19. It is clear that coordinating public health crisis response activities in the real world and online is paramount, and should be a top priority for all health care systems. We need to build more national and international detection and surveillance systems to detect the spread of infectious diseases and combat the fake news that is usually accompanied by these diseases.

## Appendix

Multimedia Appendix 1 Latent Dirichlet allocation output.

Multimedia Appendix 2 Word cloud.

Multimedia Appendix 3 Associated terms for each topic.

Multimedia Appendix 4 Descriptive statistics for sentiment and interaction analysis.

## Abbreviations

API: application program interface
COVID-19: coronavirus disease
LDA: latent Dirichlet allocation
