# Supplementary material for: Top Concerns of Tweeters During the COVID-19 Pandemic: Infoveillance Study
Source: J Med Internet Res. 2020 Apr 21;22(4):e19016. doi: 10.2196/19016 (PMC7175788; doi:10.2196/19016)
Supplement: Multimedia Appendix 1 [file jmir_v22i4e19016_app1.pdf]

Multimedia Appendix 1: LDA output

Topic 0:

help countri alreadi ask probabl viru immun guess allow woman medic expect crazi lyme poor notic weak moment servic contact strong industri offer wake throat expert slow parent consid block paper refus ha dollar rid lack idea tech parasit west assist sold sore deni somewher pdchina heavi teach arm oper cowork doesnt entri oscar lockdown toilet commerci chinadaili asap centr ive fish secret erad style iron window profession honest yellow screw thai prison lebanon vomit vulner essenti prophet chao israel standard hors knee contin steal declin vitamin seed pelosi devil sentenc cv blown pharmaceut ng fee request lunch favor sh

Topic 1:

week stay mean home viru damn run weapon season final http near biolog cours dad victim self beat univers car decid pack finish close educ tryna usual celebr tea sent massiv remain provinc space dark woke ting locat hubei soul transport ahead vacat switch port station toyota react apocalyps cho transfer professor wena subject advisori mile floor boost spent mi buri scene lite led memori solar homi coronavirusupd largest tear tesla surgeri fell prep pax dean intend mission electr seoul easier breakout encourag tradit sec elonmusk koontz goodby jamia daegu lee isra demonstr union playstat fellow logo atmospher han yokohama

Topic 2:

viru http come stop eat use spread china peopl human anim thank someth sure youtub tell live fear state say doctor old mani pandem heard kill girl happi bro caus thing game seen releas die lmao ill insid wuhan order let truth pretti question predict wipe natur yesterday wild today control mention unit bird dirt folk gave lot small recov earth consum polic biggest age kind fact forc came taken dirti detect report ye burn cool big exampl nurs prove domest evil treat deep wife mother friday pangolin sin cow nd flesh track recent innoc main kerala societi publish ago

Topic 3:

drink beer someon http read symptom viru wors student night best bottl light surviv articl know wtf took phone men song listen hide pictur modelo period leader unless infecti democrat beach bud tbh photo lung imag sweet text colleg invit tequila coke tabl rhythm premier terrifi capabl glass judg td content milk materi dress syndrom anxieti alot plenti lectur facemask cnni loud ginger relationship journal thingi relev reserv winner incred slap republican pour background pub aoc chase budweis chest grupomodelomx rapid coronarviru ali toronto slice bot difficulti soda foo bundl wire op exam donaldjtrumpjr caption tattoo rescu welp shite stella

Topic 4:

fake nah viru solv challeng killer hat mobilepunch pastor mistak gdp hoax poverti mood ang mail aye southern cc magnitud haram congrat unusu midst hv grave unemploy pa evacue section pork sacrific bound dutert ck devic uyghur oop payment ratio inteldotwav qtrresearch warren boko kya champion ufc coronaetern conflict inciner drew fare chei jet crawl cyber nick soar anchor maker malwar anthoni roast

menac coronavchart upsid coronavirusph combo scottmorrisonmp muhammad benshapiro putin knack hacker ing approxim coal cybersecur clarif fabric doe philip michel andi necessarili infant nascar rosi abscbnnew pace suspens veteran weirdo ethiopian ufo navi pti oral morrison ink

Topic 5:

time viru need know http talk believ hit dont like chang issu american tweet say water readi link spread pl hey babi movi polit handl mad tell ladi alon tonight hn tast everywher town yall faster account store join trust chicken dumb activ tf attent remov somebodi coffe piss knew stage potu tongu press mix bear abt piec sometim kick contagion confer requir birthday favorit chinacoronaviru door tax teacher nearli page statement obvious cash bite paid vietnam juic spit realis cheer urgent coverag garlic order strike deliveri bomb nail nonsens lemon cloth concern lesson certain excit insur qualiti bloomberg raw

Topic 6:

guy catch viru creat head bio border tomorrow sale idiot sun messag arrest sanit box warfar hk dong prefer gun roll berni gay saturday yell dri intellig ki tom fam hr depress ba expens advertis centuri cotton district shower float movement amidst aur egg curb kunalkamra chair gandhi virolog mini creation indoor ew fortnitegam survivor slide eugen walmart mute dawg bagh stadium shaheen significantli lame inca arrestanuj trynna hockey mat blunt avatar disinform nu delici viri atlanta ek intellectu ronaldo propheci ch mikebloomberg ahh nickiminaj escort rum shashitharoor gee dunk hoard sanctuari aapl mous sab ta warehous audio goo friendship

Topic 7:

diseas viru http meat nomeatnocoronaviru cure like watch god danger true china pm cancer deadli ji tv increas rampal saint far came maharaj aid kill sadhna saw world worship provid channel absolut avoid risk bless way hundr battl market devot oil viruscorona staff heinou eater daili sadhana necessari incur sat satsang diet bowl otherwis depend ulcer alcohol sant malaria spiritu environ saintrampaljim heineken sex listen pro intox imposs vegan drone satbhakti therefor tuesday bhakti jim father elimin patron homeless colleagu follow billion suprem miseri leadership sector improv whenev clown fb ndtv wrote whatev jagatguru ordinari havoc relief consumpt entertain diego

Topic 8:

got viru cough test friend http school ya airport morn offic conspiraci definit agre theori solut neg figur son nose dream kiss board chill queen answer potenti resid nasti monday ministri distract anybodi drunk interview facebook russia disappear york gotten suspend weed boat nyc spring nh accident wise annoy vibe german lucki rain lago typic chinatown stare ceo overcom snow remedi uae seventeen reschedul idc fucker badli boomer goin edg racoon obama subscrib guidelin zimbabw chees cream saudi threw pool gettin row lospollostv gunna sail wisdom taal scaremong cooki infront goddamn pun centenni spam aww whisper pod arabia sunset jealou

Topic 9:

viru peopl flu china make infect world die death http joke better actual kill countri india protect report spread racist number ebola rate hear popul million wuhan know quarantin govt awar citizen ban eye person deadli cover shot child suppli reach ignor scari fatal sen doctor influenza mouth base street low power especi toll instead live worldwid surpris mortal coronarivu enjoy master breath swine rise contagi

coronaawarebyvijayfan entir epidem pmoindia narendramodi welcom tag request lead healthcar london vote coronavirusindia smoke war bed philippin airborn room thing bird militari jan larg extrem reveal chain delhi coronaviruswho germani rest deliv stat perhap

Topic 10:

viru chine hope realli sick lol gonna away lime like im soon place bc http lot bitch mind die nigeria okay went hard thing caus brother rn sit tho concert postpon accord race evacu communist everybodi jesu cri pick destroy restaur shop shake came heat ground swear wit demand hurt live wont order fair star convinc smell didnt logic hair mobil ball felt pre scream broke send af legit finna yr accus accid busi artist eh uber gp equal impress mall coronavirusu christ guarante ear pc manila cu comedi squad condit prior rude deadass caa turkey jail pair stfu barcelona

Topic 11:

fuck asian yeah imagin korea goe outsid meme class fli isol climat weather worker sa north shoot lysol beauti korean danc hype discrimin fuckin multipl person western translat xenophobia sure grab festiv shirt xenophob extend aka sake indonesian sip curiou keemstar bastard eminem purchas dust replac regardless pussi exposur asshol modern zika ethnic drama hoe famou bucket femal pr circul specialist lad refresca asf coin lmaooo casual victori ngl um sol plate cigar arent reportedli showcas concept peep mosquito fart lewisham asteroid handshak toy core hm wasnt rob liquor classmat weaker sink illustr joint rli panda jamaica rank utter noon

Topic 12:

http coronaviru viru case ncov new novel covid face video virus confirm updat china coronavirusoutbreak problem deal th threat fact understand prepar suspect miss big uk feb infect amp februari latest chanc includ carri extra announc set respiratori info refer condit ice bruh advic break brand someth gov add watch summer se pull sever jump wuhancoronaviru coronaviruswuhan hai plz tokyo share concern tip fix cdcgov explain tackl mwc type amid counti beij ka coronavirustruth rd pig combat kindli daili insan apart eu winter coronaroviru outta coronaviriu zone whatsapp bewar appli deepstateexpos forev earthquak youhadonejob myth rang acut blog partner leg

Topic 13:

right fight travel chine presid stupid hong treatment anti kong claim excus nigga african quit http togeth yo protest gotta rest kobe honestli mar direct del china region focu holiday taiwan daughter regim tourist cultur privat officialefcc express resist feng bryant fong tourism lemis mainland egypt launch card bare capit art choo scientif cur spoken christian zhing jiehang shaici flue polici cap teng prioriti bother abus ran funer abeg gather focus rice david safeguard dread ring platform michael laid dose gianna skip neck ne blockchain brass ea pacif harder roundup gatewaypundit khomeinist sight passport dam tariff exit server berniesand protestor

Topic 14:

viru life mani noth http follow kid avoid post like twitter sneez save lab comment result hot come rt appar constitut list know coronaoutbreak normal cnn chloroquin bt omg kpop lost ff god repli gain footbal worth foot stan gc nsfw au human promo view prayer trick design grand fancam gg sugar furri scienc technolog dem armi donat daddi leak kenyan ruin loyal smut truli impeach olymp stray mum

ariana spot mommi bdsm gainszn chief loyali fl txt shawn citizentvkenya imma mend match tbz rp earn liber director penni aw bye agenc mutual counter sub delet sponsor tik cup tok

Topic 15:

like look viru year http pray pas relat iran stand compar drop new crown plane past recent given data basic weekend mutat term lt alcohol round patent zombi spray surfac identifi ga coz spanish bullshit forward vi damag easili ima program apolog church addit scam karma proper decad silver agent ani surround scale project pharma ride deport shape legal cocain birth renam ft begin oversea album wednesday justifi bushfir altern scenario whilst campu describ ju comput inspir clorox blind foundat mailonlin microscop independ spain def ass ktv bullet duti satellit sympathi fate iri spin slaughter electron resolv supplier auto mankind

Topic 16:

mask wear air chan stori http nobodi fall rahulgandhi peac glad misterantibulli wing blue spell park islam germ heal bank crash proud spectatorindex surgic republ central politician librari hysteria gift taco bore shoe credit quiet neighbor sky amitshah precautionari height asid allergi pretend wet boom coron mp boyfriend properti tragedi lick select harvard democraci slip epicent upcom florida punch corp male en attach cycl journey ig coronahellisen visitor bori manipul delhipolic tube correctli chri torus rahul condom instal monster flower johnson basketbal brilliant anger taxi xx cruel pt index mil territori filthi afternoon speaker den secpompeo humbl weight pal irl

Topic 17:

good work shit viru care ship cruis japan dead hate compani http lie walk say passeng line pay la special japanes stuck canada bout appl quickli despit purpos doubl quick email diamond amazon road cut malaysia bu involv respect resourc appreci shout silenc ni wast radio hashtag yea howrout crew loo aint jack edit dat gon rich ko sudden eventu european dip ny titl simpson network religi trash letter cambodia defens thursday separ gross coast yup era cunt ugh toxic angri dock session abi lowkey regist royal paul instead cast selfish secretari atm breakfast wore divis pokimanelol vape integr fella

Topic 18:

anyon effect respons racism job viru coronaviruschina local http commun mr remind cat ca fund view critic coronancov januari ccp shock simpl aliv suit john associ bag min genet kit laboratori coronaviu dm xhnew wave glove label approach stomach drericd honey bloodi closer obviou netflix depopul shown bath horror coronavirusuk congratul hunt inflat incom hazmat amen italian whistl violenc hack flew filipino jackposobiec brutal riot bay insight garbag revolut ultim voter visual legend irrespons reflect proceed acid applic antiviru coronaviruscanada procedur highlight breed conserv motiv mari blower disagre proactiv athlet therapi su learnt actor nowaday stormisuponu virtual asim inhuman maldiv

Topic 19:

viru think like man feel worri scare bad thing chine long bat told real anyth el point import dr rememb ok sound world pakistan contract stuff attack plagu caught gener shut continu matter super china laugh train speak heart ad clean kinda mass mf food healthi europ whatev indonesia pain grow mexico brexit disgust young lord er congress soup held mostli sunday clearli suddenli admit english bigger chanc everyday energi leagu stress antidot crap written hang poison variou intent iphon bulli bubbl fashion

liverpool physic mike featur draw snake wrap kingdom trust headach farm hero bbi nervou alright fresh frighten

Topic 20:

amp day viru today kill famili peopl bring month http alway think food wrong anoth white research sad black na china fast second locust clear worst known tour creatur kenya member thing later idk gate death earli huge dey anymor mexican blood sleep bought experi histori straight longer cross futur appear wide headlin stick profit simpli collect random henc coronaviruskenya beg excel ate si stone episod flesh code uni uncl tiktok com spike nomeatnocoronaviru finger shelter technic approv rel incid prob btstwt protein loona med opposit upset comeback qanon filter una narr color chernobyl gene explan broom ooo argument chip

Topic 21:

middl propaganda em fulli terror choic distribut canadian speakerpelosi angel stronger introduc score minor method lo zlj dengu vivekagnihotri complex owner assur aajtak fat tactic awa uighur scan sander competit chose remark wat vehicl hint incindia programm assembl hatr android bang manutd diver vile bf dashboard yellowcorona mere butt luv genocid hurrican cheat isi dj imtiazmadmood sundaythought multipli innov draft bean equival bhakt divid retir ole radic cannabi gender stereotyp peanut queue nhsuk shield wuhanlockdown jose coronarobot imran airway nvm fluid il mfachina pitch cm attribut outlook biggboss deceas inspect bait jay introduct withstand dnc birdflu ilhanmn commod ighalojud rippl

Topic 22:

viru god happen cancel http eat china great flight quarantin free everyth close break suffer plan book word market wish usa leav trade muslim virus start time sell meat law forget trip singapor fine sin forc refund fan vegetarian war ticket st valentin terribl form human follow thailand precaut organ becom camp step team march east nomeatnocoronaviru correct big command suck intern rule airlin consequ holi punish snake improv victim hello charg respond demon aris illeg opportun hotel calm foreign seri reaction indic paranoid financi id fed australia unlik twice contrari spi senat concentr freedom schedul loss valu airasiasupport volunt

Topic 23:

viru http outbreak china tri trump thought mayb wait think oh realdonaldtrump wonder medium check affect know reason play ye africa buy busi high money funni america littl spread contain indian stock support posit origin afraid event panic market treat wuhan concern dude south gone warn asia product area win ago scientist nice drug delay late dear wow far anoth social real till arriv coupl minut medic similar land googl suppos discus major live order custom onlin current plu discov april screen safeti amaz attend team rais connect best billion throw storm search practic facil confus engin write produc carrier

Topic 24:

viru want said way start http caus everi hospit cold sar seriou citi wuhan differ corrupt gt wanna common new blame strain boy meet price medicin sourc hell learn complet cuz date type trend red allah pop studi deserv expo california alert pneumonia singl exactli record mer china easi coronavirus weird rip princess perfect music assum cdc magu thread bos wuhanviru cov pizza freak built earlier serv

topic click shortag bacteria cute model nytim superbowl smart combin individu chinaviru disinfect complain cheap doc epidem access voic besid went pretti observ sing skin tough maga review strongli benefit base award efcc

Topic 25:

health vaccin patient prevent public viru possibl nation risk control le offici sorri hiv outbreak develop total current emerg http parti measur fun infect crisi group kind higher half manag regard diagnos author minist avail bar brain note count level zero doubt ww host transmiss declar wall novel immedi cost nigerian stream transmit gold depart accept promot mental number site institut clinic drtedro promis panick employe administr sequenc collaps enemi thu hygien statist genom awesom confid organis dna trial conduct ralli prepared margarita coronabeeru repres progress quarter role jersey committe feder ensur iraq ard discharg abil bond destin senior rna

Topic 26:

love dog build abl viru opinion mess orang btw podcast websit british pet plant veg crack hug fault lil seat moon lmfao tune prime plot valentinesday corpor profil useless sensit pump tree whitehous guest tap cgtnoffici robot wed desper wan reject metal dozen phase soft slightli washington washingtonpost path mountain squar dailyn troll thehil dig infrastructur wth umbrella lunar septemb billi colour venu sonic reliev chai snack duh collabor skill leftist abort raccoon preciou dept disgrac bash anagram admin phillip rocket arizona coronaextrausa realanuj franci commi schofield icon chaosincorona fawadchaudhri authoritarian nt jordan jo hum tournament plead boyl netherland spotifi

Topic 27:

smh haha sister drive version forgot sampl fav ugli tortur contribut print ciara caution distanc timelin flat tu dettol whowpro pint forest humor ajenglish whoafro gf mcdonald whosearo signal corononaviru taught whoemro goat ish socialist covidnew desert whoeurop repent cloud nz render pahowho bullish swing agreement certif flyer convert breakingnew flavor adapt plug merch purg bitter scmpnew boot vax stockmarket alessiocorona insist banana toosoonforcoronavirustun sana asham workplac beast caribbean leaf fandom leo chartdata ncdcgov drove loop satan menu wk nike coronapyramoa workshop harsh misspel aswel lah someday accent ross ada sane ling defo dev cam prosecut jerk kh kudo senschum

Topic 28:

viru plea news say govern everyon http let hand sinc end peopl inform china stop person ppl open make hous turn bodi ur send mom wash becom visit hour scienc non enter touch bit australia share mani disast knowledg thousand epidem fail act left short save hold present affect bet begin suggest manufactur elect leav realiz san sign spiritu live pakistani specif unabl cover struggl spend action shame packag film mate result sort itali secur planet contact infect listen escap recommend player heard type internet realjameswood invent bjp contamin empti chat defeat murder power odd feed avert goal franc advanc

Topic 29:

safe global situat economi viru impact liter http fever viral sir hi return econom exist brought receiv warm ah lock flood drank pic bitcoin factori initi invest vodka growth mark lassa load monitor club yang

inde budget crypto china analysi hahaha option recess size cousin bleach drsjaishankar genuin shanghai ai msm btc audienc investor evolv extinct gym french hmmm engag bush gang pose rout censor river coronviru appoint bhi arvindkejriw disney calam npr lit nba inevit percentag sentiment mohkenya hardli linkedin surg nrc cabin nahi hurri crude sri vaxxer recogn congo santa effici sheep uncertainti mainstream pant poll aircraft vietnames
