# Supplementary material for: Top Concerns of Tweeters During the COVID-19 Pandemic: Infoveillance Study
Source: J Med Internet Res. 2020 Apr 21;22(4):e19016. doi: 10.2196/19016 (PMC7175788; doi:10.2196/19016)
Supplement: Multimedia Appendix 3 [file jmir_v22i4e19016_app3.pdf]

Multimedia Appendix 3: Associated terms for each topic

Topics	Selected Search Terms
China	china, chines, wuhan
Outbreak	outbreak
Eating Meat	meat, eat , eating
Developing Bioweapon	bio, weapon
Fear & Stree of COVID-19	fright, horror, panic, scare, terror, fear, concern, stress, tension, strain, alarm, denger, danger
Deaths caused by COVID-19	dead, died, kill, death, sick, sneez, caugh, patient, ill
Travel Bans and Warnings	travel, flying, flight, cruis, ticket
Panic Buying	panic buy, product, sold out, toilet, store, sell , bought
Economic Losses	econom, product, company, factory, business, financ
Increase Racism	racist, racism
Quarantining Subjects	quarantin
Wearing Masks	mask
